# Drak2 Does Not Regulate TGF-β Signaling in T Cells

**DOI:** 10.1371/journal.pone.0123650

**Published:** 2015-05-07

**Authors:** Tarsha L. Harris, Maureen A. McGargill

**Affiliations:** 1 Department of Immunology, St. Jude Children’s Research Hospital, Memphis, Tennessee, United States of America; 2 Integrated Biomedical Sciences Program, University of Tennessee Health Science Center, Memphis, Tennessee, United States of America; University of Münster, GERMANY

## Abstract

Drak2 is a serine/threonine kinase expressed highest in T cells and B cells. *Drak2^-/-^* mice are resistant to autoimmunity in mouse models of type 1 diabetes and multiple sclerosis. Resistance to these diseases occurs, in part, because Drak2 is required for the survival of autoreactive T cells that induce disease. However, the molecular mechanisms by which Drak2 affects T cell survival and autoimmunity are not known. A recent report demonstrated that Drak2 negatively regulated transforming growth factor-β (TGF-β) signaling in tumor cell lines. Thus, increased TGF-β signaling in the absence of *Drak2* may contribute to the resistance to autoimmunity in *Drak2^-/-^* mice. Therefore, we examined if Drak2 functioned as a negative regulator of TGF-β signaling in T cells, and whether the enhanced susceptibility to death of *Drak2^-/-^* T cells was due to augmented TGF-β signaling. Using several *in vitro* assays to test TGF-β signaling and T cell function, we found that activation of Smad2 and Smad3, which are downstream of the TGF-β receptor, was similar between wildtype and *Drak2^-/-^* T cells. Furthermore, TGF-β-mediated effects on naïve T cell proliferation, activated CD8^+^ T cell survival, and regulatory T cell induction was similar between wildtype and *Drak2^-/-^* T cells. Finally, the increased susceptibility to death in the absence of *Drak2* was not due to enhanced TGF-β signaling. Together, these data suggest that Drak2 does not function as a negative regulator of TGF-β signaling in primary T cells stimulated *in vitro*. It is important to investigate and discern potential molecular mechanisms by which Drak2 functions in order to better understand the etiology of autoimmune diseases, as well as to validate the use of Drak2 as a target for therapeutic treatment of these diseases.

## Introduction

T cells play crucial roles in tumor surveillance and protection against invading pathogens. However, if not properly regulated, T cells can attack normal healthy cells of the body. This defective response may lead to tissue destruction and devastating autoimmune diseases such as type 1 diabetes and multiple sclerosis. Treatments to modify the progression of autoimmune diseases often include immunosuppressant medications that lead to enhanced susceptibility to infections and tumors. Inhibition of Drak2, a serine-threonine kinase, may be an alternative approach to inhibit autoreactive T cells without acting as an immunosuppressant.

*Drak2* ^*-/-*^ mice are resistant to autoimmune disease in mouse models of type 1 diabetes and multiple sclerosis [1,2]. In both of these disease models, the accumulation of autoreactive T cells in the target organ is significantly reduced in the absence of *Drak2*. The reduced accumulation of autoreactive T cells is due to an increased susceptibility to death of the *Drak2* ^*-/-*^ T cells [2,3]. Interestingly, despite this increased sensitivity to death in the T cells, the *Drak2* ^*-/-*^ mice effectively eliminate infectious pathogens and retain the ability to combat tumors as well as wildtype mice [2,4–7]. Thus, Drak2 is an ideal protein to target in order to treat autoimmune disorders without compromising immunity to pathogens and tumors. However, the substrates and downstream effects of Drak2 signaling that contribute to autoimmunity require further elucidation to validate its potential as a therapeutic target and to further understand how these autoimmune diseases develop.

Drak2 has been shown to interact with several proteins in *in vitro* recombinant assays and in cell lines. These proteins include myosin light chain [8], calcineurin homologous protein [9], Protein kinase D [10], p70S6 kinase [11], and TGF-β receptor I (TGF-βRI) [12]. However, most of these interactions have not been confirmed in T cells and therefore, it is not clear which of these interactions may affect autoimmune disease.

As TGF-β is a critical suppressor of autoimmunity, the interaction of Drak2 and the TGF-βRI is an intriguing possibility to explain how Drak2 contributes to autoimmunity. TGF-β is a pleiotropic cytokine that elicits numerous effects on various cell types [13]. In T cells specifically, TGF-β inhibits proliferation of naïve T cells, induces development of regulatory T cells, and enhances apoptosis of activated T cells. A recent study proposed that Drak2 functions as a negative regulator of TGF-β signaling by inhibiting the phosphorylation and recruitment of Smad2 and Smad3 to the TGF-βRI in cell lines [12]. Thus, the absence of *Drak2* in T cells may render these cells more susceptible to TGF-β signaling, which could prevent autoimmunity. However, it has not been tested if Drak2 functions as a negative regulator of TGF-β in T cells, and consequently, whether *Drak2* ^*-/-*^ T cells are more sensitive to TGF-β signaling.

Therefore, we investigated whether Drak2 functions as a negative regulator of TGF-β signaling in T cells, and further if the enhanced susceptibility to apoptosis in *Drak2* ^*-/-*^ T cells was due to augmented TGF-β signaling. We found that TGF-β signaling via Smad2 and Smad3 was not enhanced in the absence of *Drak2* in T cells, and that *Drak2* ^*-/-*^ T cells did not exhibit enhanced responses to TGF-β signaling during *in vitro* assays. These data suggest that Drak2 does not function as an inhibitor of TGF-β signaling in T cells. Moreover, in the absence of TGF-β signaling, *Drak2* ^*-/-*^ T cells remained more susceptible to apoptosis, suggesting that the increase in cell death observed *in vitro*, was not due to enhanced TGF-β-mediated signals. These data provide insight into the role of Drak2 in autoimmune diseases by showing that Drak2 may not suppress TGF-β signaling in T cells, and therefore may contribute to autoimmune disease via other molecular pathways.

## Materials and Methods

### Mice

*B6*.*Drak2* ^*-/-*^ mice were previously described and backcrossed 19 generations to C57BL/6 [1]. *OT-II* mice were obtained from Kristin Hogquist, *TGF-βDNRII* mice were obtained from Hongbo Chi, *C57BL/6*, *CD451/1*, and *OT-I* mice were purchased from Jackson Laboratories. Mice were held under specific pathogen-free conditions at St. Jude Children’s Research Hospital.

### Ethics Statement

All studies were reviewed and approved by the St. Jude Animal Ethics Committee under protocol number 486-100303-05/14. St. Jude is AAALAC accredited and complies with all federal, state, and local laws.

### FACs purification of lymphoid populations

T cells were purified from the spleen and lymph nodes of mice by FACS sorting with antibodies specific for CD4, CD8, CD25, CD44, and CD62L (eBioscience). Naïve T cells were CD25^-^CD44^lo^ or CD25^-^CD44^lo^CD62L^hi^. Cell sorting was performed using the iCyt Reflection or SY3200 Cell Sorters (Sony Biotechnology).

### Magnetic separation of CD4^+^ T cells

T cells were purified from the lymph nodes of mice by negative selection with biotin-conjugated antibodies specific for B220, CD8, CD11b, DX5, and MHC class II (eBioscience), followed by separation with streptavidin-conjugated magnetic beads (Miltenyi Biotech).

### Flow Cytometric Analysis

Single cell suspensions from *in vitro* cultures were stained with antibodies specific for CD4, CD8, CD25, CD45.1, and CD45.2 (eBioscience and BioLegend). Cells were analyzed on a FACSCalibur or LSRFortessa (BD Biosciences,). Cell death and viability was determined utilizing Annexin V (BD Biosciences) or Fixable Viability Dye (eBioscience), according to manufacturer’s instructions. Analysis was performed with FlowJo software (TreeStar, Inc.). To detect Foxp3^+^ cells, suspensions were stained with antibodies specific for CD4, CD8, and CD25. Cells were then fixed and permeabilized with the Foxp3/Transcription Factor Staining Buffer Set according to manufacturer’s instructions (eBioscience) and stained with anti-Foxp3 antibody (eBioscience). For analysis of phosphorylated Smad2/3, cells were stained with antibodies specific for CD4 and CD8, then fixed with 1X BD Phosflow Lyse/Fix Buffer and permeabilized with BD Phosflow Perm Buffer III according to manufacturer’s instructions (BD Biosciences) and stained with anti-pSmad2/3 antibody (BD Biosciences).

### Plate-bound anti-CD3 and anti-CD28 stimulation

Tissue culture-treated plates were incubated for one hour with 30μg/ml goat anti-hamster IgG in PBS (Vector Laboratories, Burlingame, CA), then washed and incubated for one hour with 1μg/ml or 2μg/ml anti-CD3 (eBioscience). Plates were washed before addition of cells and 1μg/ml anti-CD28 (eBioscience).

### Stimulation of *OT-I* cells

Naïve *OT-I* CD8^+^ T cells were sorted and labeled with 5,6-carboxyfluorescein diacetate succinimidyl ester (CFSE) (Molecular probes) at 0.4 μM in pre-warmed PBS (0.1% FCS) for 10 minutes at 37°C, then washed twice with RP10 advanced media (RPMI advanced media, 10% FCS, Hepes, Pen-Strep, L-glutamine, BME, gentamicin). Cells were stimulated *in vitro* for two days with 100pM OVA_257_ peptide-pulsed, CD45.1 splenocytes that were irradiated at 3000 rads.

Alternatively, 2 x 10^6^ FACS-sorted, naïve *OT-I* CD8^+^ T cells were stimulated with 4.5 x 10^6^ 200 nM OVA_257_ peptide-pulsed, CD45.1 splenocytes at 37°C for two days in RP10 advanced media. The cells were harvested, washed, and replated with naïve splenocytes in the presence of 5ng/ml TGF-β (R&D Systems) with or without 20 ng/ml recombinant mouse IL-2 (BD Biosciences), IL-7 (Invitrogen Life Technologies), or IL-15 (R&D Systems). Two days later, fresh media and cytokines were added, and two days later, cells were harvested, stained and analyzed by flow cytometry.

### Stimulation of *OT-II* cells

Naïve *OT-II* CD4^+^ T cells were sorted, CFSE labeled, and stimulated *in vitro* for three days in the presence or absence of 10-fold TGF-β titrations with 10μM OVA_323_ peptide-pulsed, irradiated splenocytes. After three days, the cells were harvested and analyzed by flow cytometry.

### *In vitro* Treg induction

Naïve CD4^+^ T cells were purified and stimulated with plate-bound anti-CD3 and soluble anti-CD28 for 72 hours with 20ng/ml IL-2 and increasing amounts of TGF-β. Cells were analyzed for Foxp3 expression.

### Fluorescent microscopy

Wildtype and *Drak2* ^*-/-*^ CD4^+^ cells were negatively selected with Miltenyi beads and stimulated with 1μg/ml anti-CD3 coated on poly L-lysine-coated coverglass slides and 1μg/ml soluble anti-CD28 for 24 hours. TGF-β was added to some samples for the final 20 minutes. Cells were fixed with 4% methanol-free formaldehyde, permeabilized in 0.1% Triton-X in PBS, washed with PBS, blocked with 1% BSA, and incubated with anti-Smad2 antibody (Cell Signaling) overnight. Cells were stained with Alexa Fluor 647 goat anti-Rabbit, Alexa Fluor 488 Phalloidin, and DAPI (Invitrogen Life Technologies). Images were collected utilizing a Nikon C1Si laser scanning confocal microscope.

### Western Blot analysis

Spleen and lymph nodes were harvested from wildtype and *Drak2* ^*-/-*^ mice. Whole splenocytes and FACS-sorted naïve CD4^+^ and CD8^+^ T cells were stimulated for two hours with plate-bound anti-CD3 and anti-CD28 with or without 2 ng/ml TGF-β for one additional hour. Cells were harvested and frozen at -80°C. Frozen cell pellets were lysed (50mM Tris, 150mM NaCl, 1% Triton X-100, 0.5% sodium deoxycholate, 2mM EDTA, 10% glycerol with phosphatase and protease inhibitors (Calbiochem). Protein concentration was determined using a BCA Protein Assay (Thermo Scientific). Equal amounts of protein were denatured in sample buffer (10% SDS, 20% Glycerol, 0.2M Tris HCl, 0.05% Bromophenol Blue), separated by SDS-PAGE, and transferred to PVDF membranes for immunoblot analysis.

## Results

### TGF-β signaling via Smad proteins is not enhanced in *Drak2* ^*-/-*^ T cells compared to wildtype T cells

**Fig 1:**
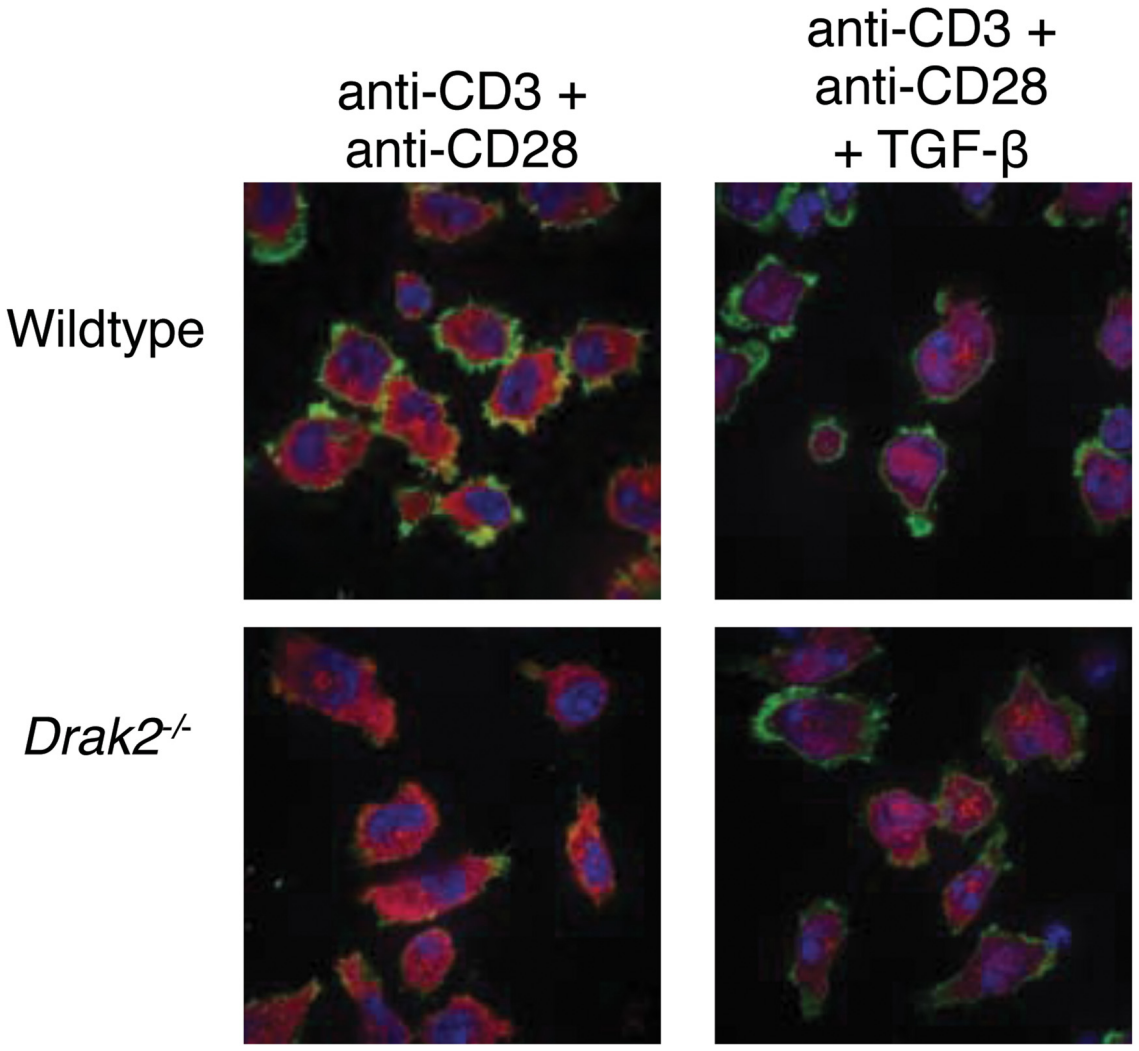
Smad2 translocation is not enhanced in *Drak2-/-* T cells compared to wildtype T cells. Wildtype and *Drak2-/-* CD4^+^ cells were negatively selected with Miltenyi magnetic beads and stimulated on anti-CD3-coated coverglass slides along with soluble anti-CD28 for 24 hours. Half of the cells were treated with TGF-β for the final 20 minutes of culture. Cells were fixed, permeabilized, and stained with DAPI, Phalloidin, and anti-Smad2. Images were collected via confocal microscopy. n = 2 mice per group. Data are representative of two independent experiments.

Given that recent experiments in cell lines suggested that Drak2 negatively regulates TGF-β signaling [12], and enhanced TGF-β signaling in T cells could contribute to the resistance to autoimmune disease, we tested whether Drak2 functions as a negative regulator of TGF-β signaling in T cells. TGF-β receptor engagement results in the phosphorylation of the Smad2/Smad3 signaling complex, which then translocates from the cytoplasm into the nucleus to facilitate TGF-β-mediated transcription. To determine if Smad2 translocation into the nucleus was increased in the absence of *Drak2*, we activated CD4^+^ T cells with anti-CD3 and anti-CD28 antibodies for 24 hours, and then utilized confocal fluorescent microscopy to analyze Smad2 localization following addition of TGF-β. As expected, Smad2 translocation into the nucleus was not observed in stimulated T cells without exogenous TGF-β (Fig 1). The addition of TGF-β during the final 20 minutes of culture elicited Smad2 translocation into the nuclear region of both wildtype and *Drak2* ^*-/-*^ T cells (Fig 1). Importantly, there were no differences in Smad2 translocation between wildtype and *Drak2* ^*-/-*^ T cells in response to exogenous TGF-β.

**Fig 2:**
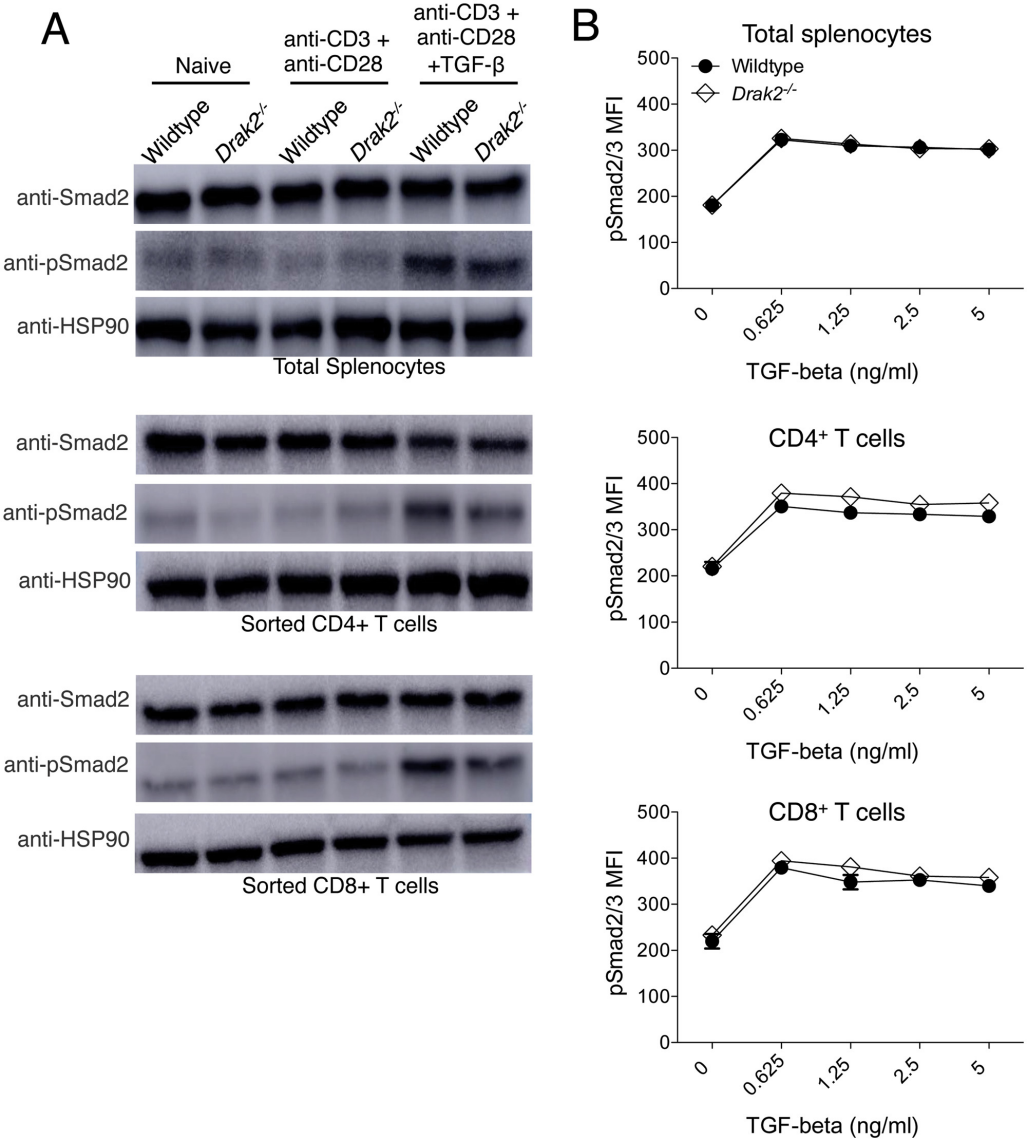
Smad2 and Smad2/3 complex phosphorylation is not enhanced in *Drak2-/-* T cells compared to wildtype T cells. A) Wildtype and *Drak2-/-* splenocytes, and FACS sorted naïve CD4^+^ and CD8^+^ T cells were stimulated for 2 hours with anti-CD3 and anti-CD28, with or without 2 ng/ml TGF-β for one additional hour. Cells were lysed and analyzed by western blot with antibodies specific for Smad2, phosphorylated Smad2, and HSP90 as a loading control. Cells were pooled from 9 wildtype and 8 *Drak2-/-* mice. Data are representative of two independent experiments. B) Wildtype and *Drak2-/-* splenocytes were stimulated for 2 hours with anti-CD3 and anti-CD28 with or without increasing concentrations of TGF-β for one additional hour. The cells were harvested, stained with antibodies specific for CD4, CD8, and pSmad2/3, and analyzed by flow cytometry. The average mean fluorescence intensity (MFI) of pSmad2/3 expression is shown for 3 mice per group. There was no significant difference in the response of the wildtype and *Drak2-/-* cells according to the Mann-Whitney *U*-test. Data are representative of 3 independent experiments.

We also examined phosphorylation of Smad2 by western blot in lysates from purified CD4^+^ T cells, CD8^+^ T cells, or whole splenocytes. In all cell types, Smad2 was phosphorylated in response to TGF-β treatment; however, the extent of phosphorylation was not increased in *Drak2* ^*-/-*^ cells compared to wildtype cells (Fig 2a). Finally, to determine if *Drak2* ^*-/-*^ cells are hypersensitive to lower concentrations of TGF-β, we analyzed the phosphorylation of the Smad2/Smad3 complex by flow cytometry in response to decreasing amounts of TGF-β. Again, even at the lower doses of TGF-β, the phosphorylation of Smad2/3 was similar in wildtype and *Drak2* ^*-/-*^ cells (Fig 2b). Together, these data show that *Drak2* ^*-/-*^ T cells do not exhibit enhanced TGF-β signaling via Smad2 or Smad2/3 complex phosphorylation compared to wildtype T cells, suggesting that Drak2 does not function as a negative regulator of TGF-β signaling in primary T cells activated *in vitro*.

### The effects of TGF-β on T cells are comparable between wildtype and *Drak2* ^*-/-*^ T cells

**Fig 3:**
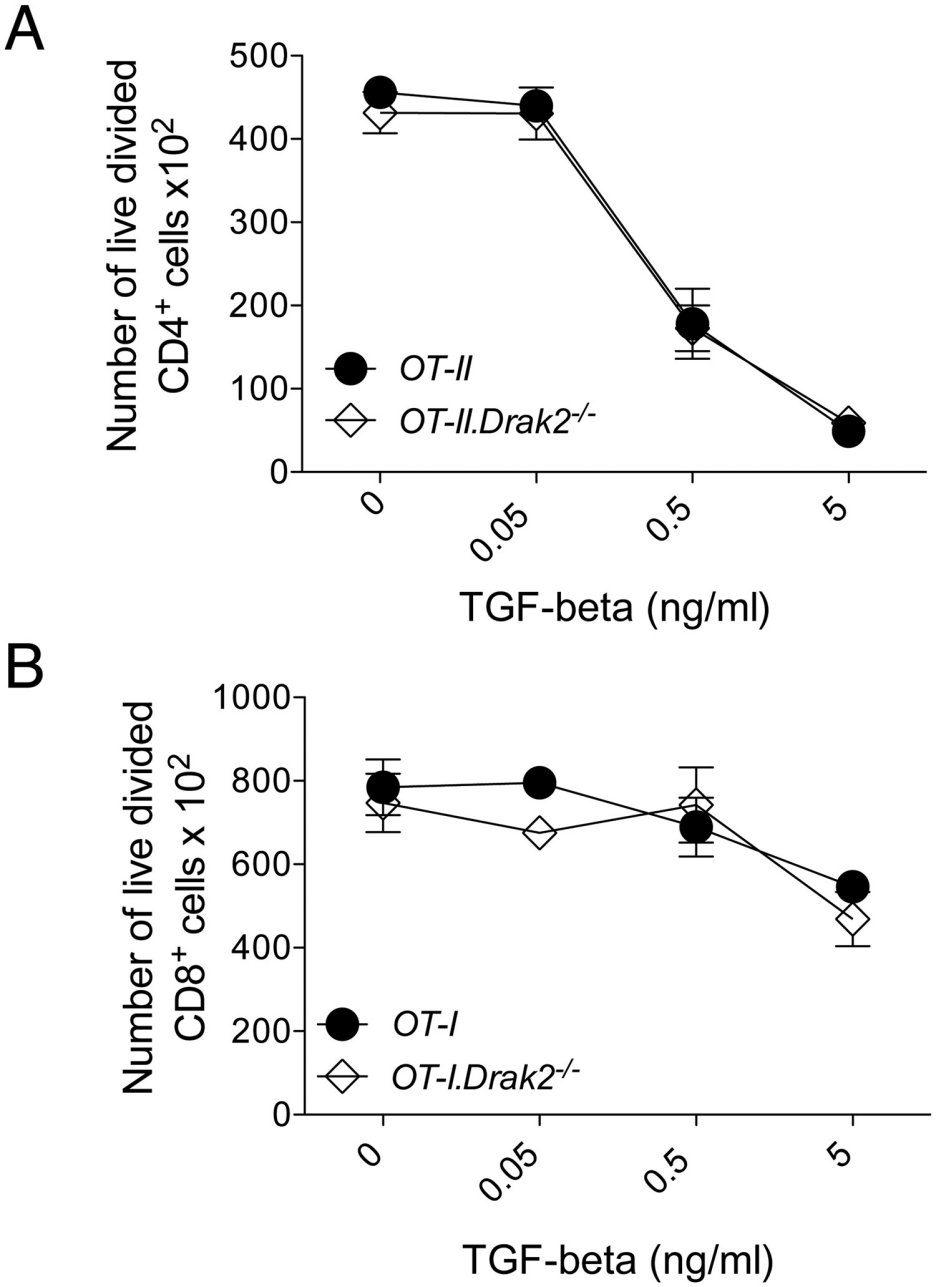
TGF-β-mediated inhibition of naïve T cell proliferation is comparable between wildtype and *Drak2-/-* T cells. A) CD4^+^CD25^-^CD44^lo^ naïve cells were purified from *OT-II* and *OT-II*.*Drak2-/-* mice and stimulated with irradiated splenocytes loaded with 10μM OVA_323_ peptide in the presence or absence of 10-fold TGF-β titrations for three days. The number of live, divided Foxp3^-^CD4^+^ cells are shown for each titration. Cells were obtained from one *OT-II* or *OT-II*.*Drak2-/-* mouse and tested in quadruplicate. Data are representative of five separate experiments. B) CD8^+^CD25^-^CD44^lo^CD62L^hi^ naïve cells were purified from *OT-I* and *OT-I*.*Drak2-/-* mice and stimulated with splenocytes loaded with 100pM OVA_257_ peptide in the presence or absence of 10-fold TGF-β titrations. Two days later, cells were harvested and analyzed by flow cytometry. The number of live, divided CD8^+^ cells are shown for each titration. Cells were obtained from one *OT-I* or *OT-I*.*Drak2-/-* mouse and tested in quadruplicate. Data are representative of three separate experiments. There was no significant difference in the response of the wildtype and *Drak2-/-* cells according to the Mann-Whitney *U*-test.

Many of the downstream mechanisms utilized by TGF-β to regulate T cells remain unclear. Although we did not observe enhanced TGF-β signaling in *Drak2* ^*-/-*^ T cells via Smad proteins, it was possible that Drak2 regulated the pathway through alternative mechanisms. Therefore, we explored the effects of TGF-β on several T cell functions *in vitro*. TGF-β suppresses T cell receptor-induced proliferation of naïve T cells *in vitro* [14]. Thus, we examined if naïve *Drak2* ^*-/-*^ T cells were more sensitive to TGF-β-mediated inhibition of proliferation than naive wildtype T cells. Naïve *OT-II* and *OT-II*.*Drak2* ^*-/-*^ CD4^+^ T cells were stimulated with OVA_323_-pulsed splenocytes in the presence or absence of TGF-β, and analyzed for proliferation. The number of live, divided CD4^+^ T cells decreased in response to TGF-β (Fig 3a). However, the effect of TGF-β inhibition was comparable between *OT-II* and *OT-II*.*Drak2* ^*-/-*^ T cells. We also tested the effect of TGF-β on proliferation of naïve CD8^+^ T cells, by stimulating *OT-I* and *OT-I*.*Drak2* ^*-/-*^ T cells with OVA_257_-pulsed splenocytes in the presence of TGF-β. Similar to CD4^+^ T cells, the number of live, divided CD8^+^ T cells decreased in response to TGF-β, and the amount of suppression was similar between *OT-I* and *OT-I*.*Drak2* ^*-/-*^ T cells (Fig 3b), again suggesting that TGF-β signaling was not enhanced in the absence of *Drak2*.

**Fig 4:**
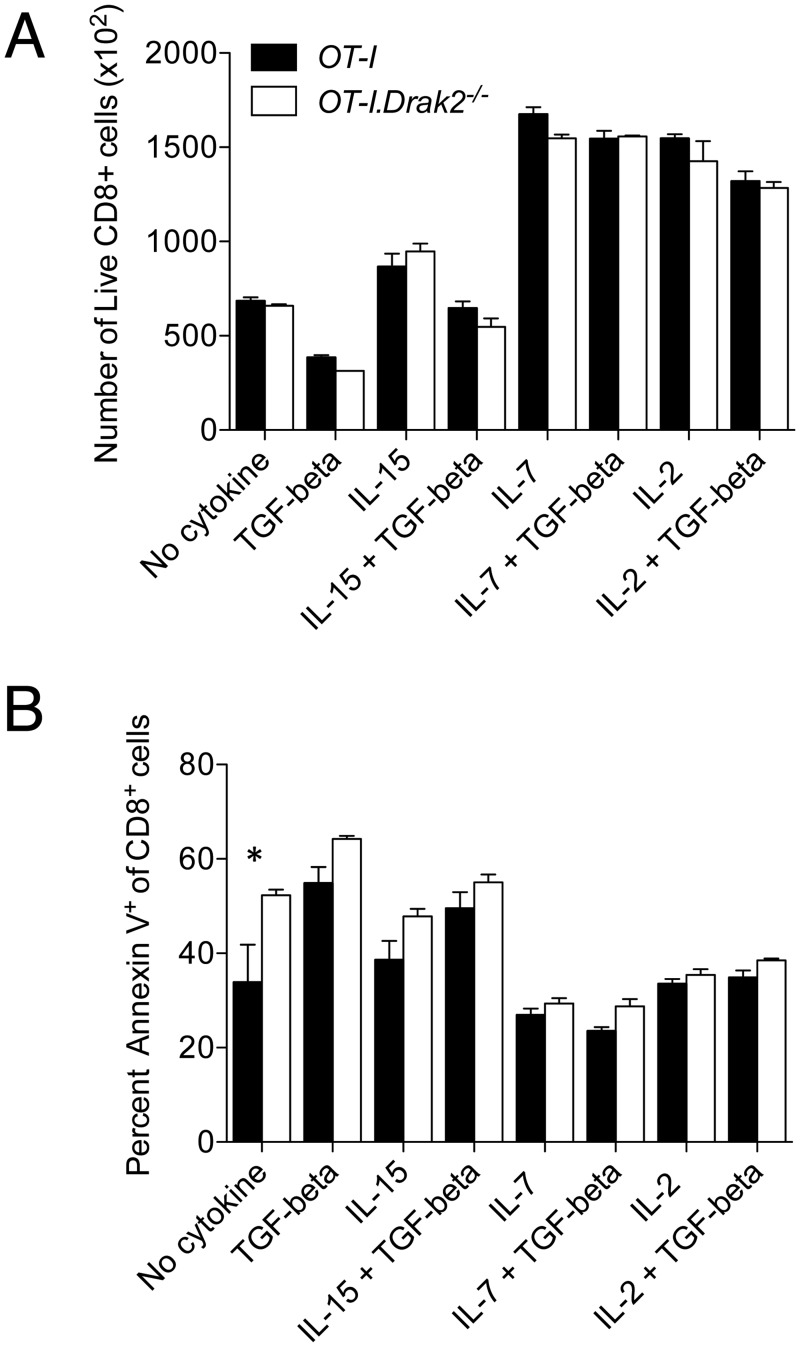
TGF-β-mediated responses to opposing cytokines are comparable between wildtype and *Drak2-/-* T cells. CD8^+^CD25^-^CD44^lo^CD62L^hi^ naïve cells were purified from *OT-I* and *OT-I*.*Drak2-/-* mice and stimulated with 100nM OVA_257_–pulsed splenocytes for 2 days. Cells were harvested and replated at equal numbers with or without various cytokine combinations. Cytokines were replenished 2 days later. Cells were harvested and analyzed by flow cytometry on day 6. A) The number of live, CD8^+^ cells and B) percent Annexin V^+^ of CD8^+^ cells are shown for each cytokine condition. Cells were obtained from one *OT-I* or *OT-I*.*Drak2-/-* mouse and tested in quadruplicate. Data are representative of two independent experiments. *P < 0.*05* (Mann-Whitney *U-*test).

TGF-β can abrogate survival signals provided by IL-15, but not those elicited by IL-2 and IL-7 in expanding CD8^+^ T cells [15]. To determine if TGF-β function in response to opposing cytokines is altered in the absence of *Drak2*, we explored the antagonistic effects of TGF-β on cell recovery and survival of activated CD8^+^ cells. *OT-I* and *OT-I*.*Drak2* ^*-/-*^ cells were stimulated with OVA_257_-pulsed splenocytes for two days. Cells were then washed and cultured with exogenous IL-2, IL-7, or IL-15 with or without TGF-β for an additional four days. The addition of TGF-β decreased the number of live CD8^+^ T cells compared to culture in media alone (Fig 4a). Adding IL-2, IL-7, and IL-15 enhanced the recovery of live CD8^+^ T cells compared to culture in media alone. The addition of TGF-β masked the increased recovery in response to IL-15, but not IL-2 and IL-7. Decreased cell recovery in response to TGF-β compared to culture in media alone correlated with an increase in the proportion of Annexin V^+^ apoptotic cells (Fig 4b). The addition of TGF-β abrogated the survival effects of IL-15, but did not alter the anti-apoptotic effects of IL-2 and IL-7. However, the ability of TGF-β to oppose the effects of IL-15, but not IL-2 and IL-7 was comparable between *OT-I* and *OT-I*.*Drak2* ^*-/-*^ T cells, suggesting that these TGF-β-mediated effects are not enhanced in the absence of *Drak2*. These data further indicate that TGF-β signaling and function is not increased in *Drak2* ^*-/-*^ T cells compared to wildtype T cells following *in vitro* stimulation.

### TGF-β-mediated T cell differentiation is not altered in the absence of *Drak2*

**Fig 5:**
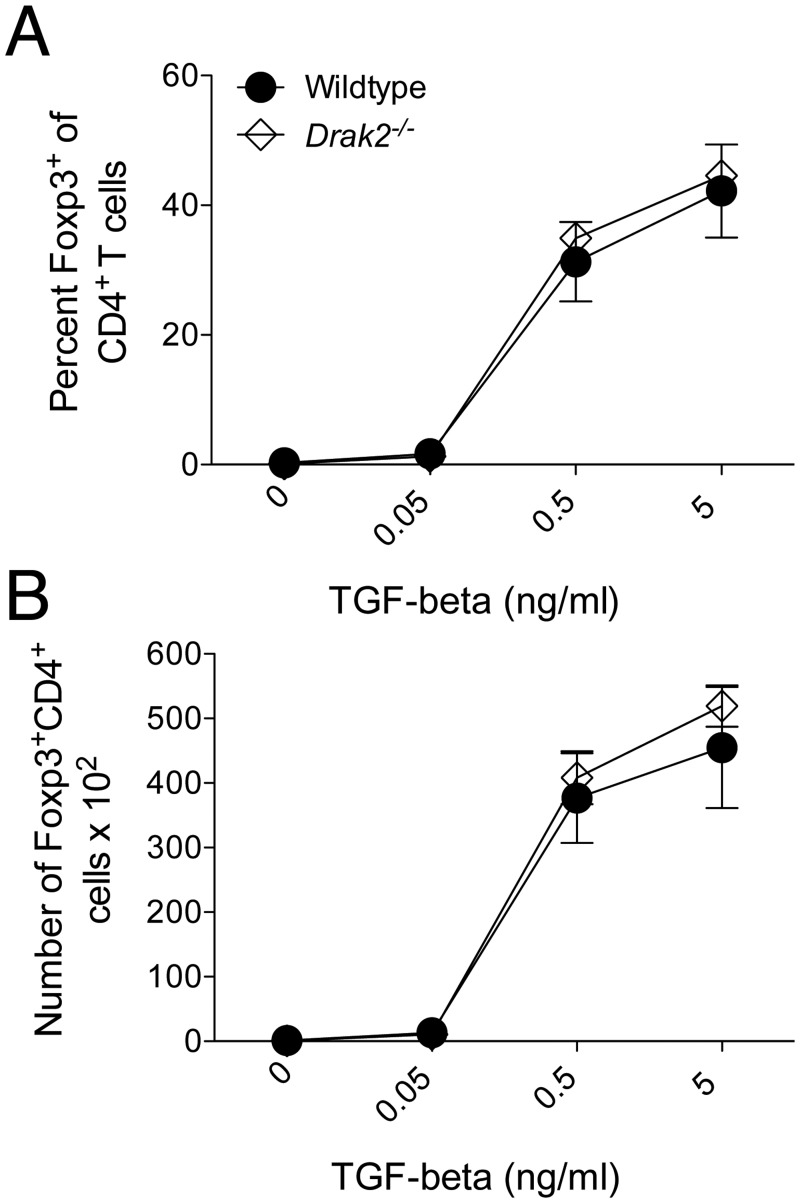
TGF-β-mediated regulatory T cell induction is not altered in the absence of *Drak2*. A) CD4^+^CD25^-^CD44^lo^ naïve cells were purified from wildtype and *Drak2-/-* mice and stimulated with 1μg/ml anti-CD3 and 1μg/ml anti-CD28 with 20ng/ml IL-2 alone or plus 10-fold TGF-β titrations for 3 days. The A) percent and B) number of Foxp3^+^ cells of electronically gated CD4^+^ cells is shown. There was no significant difference in the response of the wildtype and *Drak2-/-* cells according to the Mann-Whitney *U*-test.

Another function of TGF-β is the induction of peripheral regulatory T cells [16]. As regulatory T cells are critical to prevent autoimmune diseases, we explored if there were alterations in TGF-β-mediated differentiation of induced regulatory T cells. Naïve wildtype and *Drak2* ^*-/-*^ CD4^+^ T cells were purified and stimulated *in vitro* with anti-CD3, anti-CD28, and IL-2, with increasing amounts of TGF-β (Fig 5). The addition of TGF-β increased Foxp3 expression, indicative of regulatory T cell induction. However, we did not observe an enhanced induction in the percent (Fig 5a) or number (Fig 5b) of Foxp3^+^CD4^+^ cells in the absence of *Drak2*. These data also suggest that TGF-β functions similarly in wildtype and *Drak2* ^*-/-*^ T cells that were activated *in vitro*. Therefore, Drak2 may not act as a negative regulator of TGF-β signaling in T cells.

### Enhanced susceptibility to death of *Drak2* ^*-/-*^ T cells compared to wildtype T cells is independent of TGF-β signaling *in vitro*

**Fig 6:**
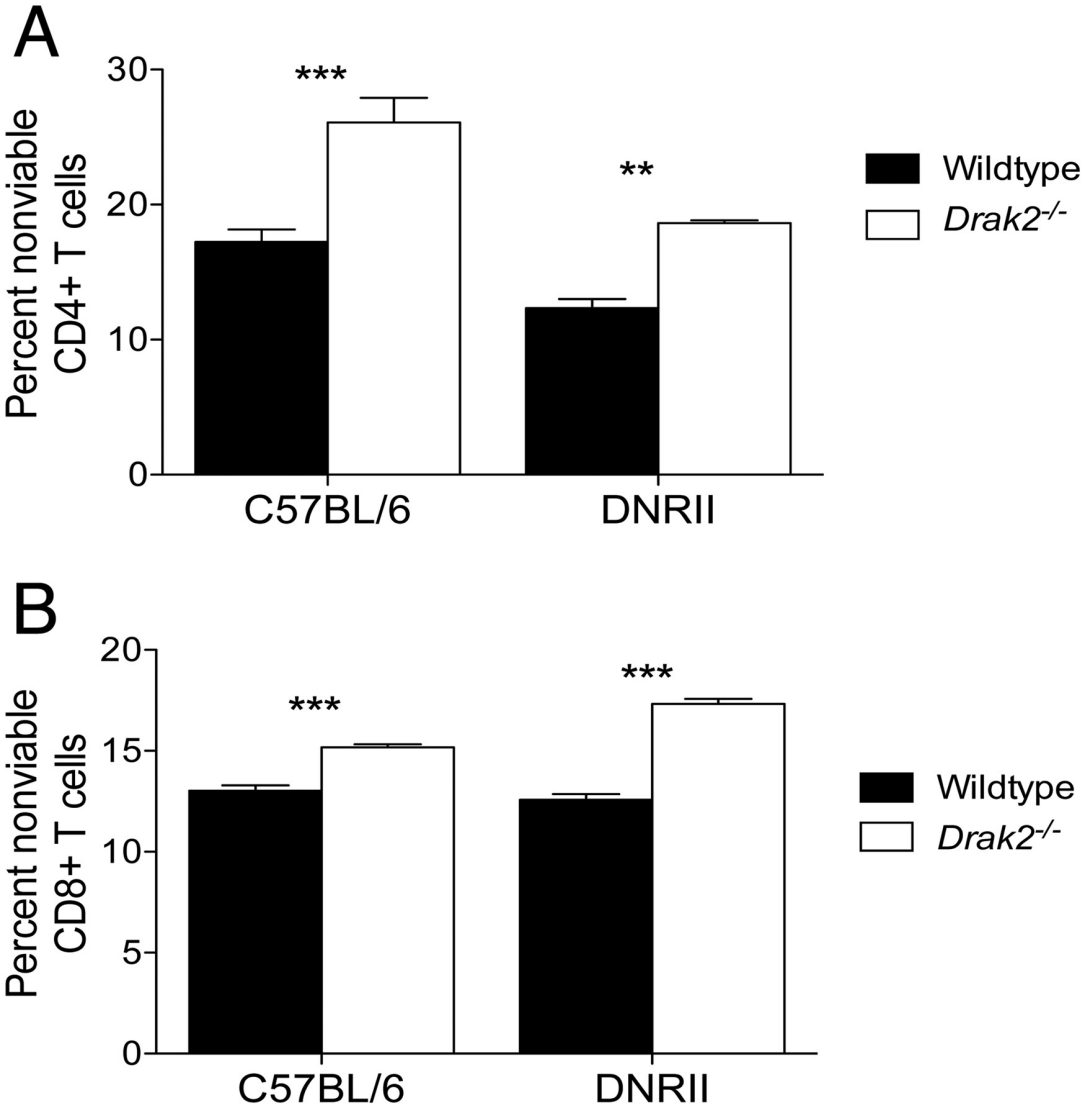
Enhanced susceptibility to death of *Drak2-/-* T cells compared to wildtype T cells is independent of TGF-β signaling *in vitro*. A) CD4^+^CD25^-^CD44^lo^ or B) CD8^+^CD25^-^CD44^lo^ naïve cells were purified from wildtype, *Drak2-/-*, *DNRII*, and *DNRII*. *Drak2-/-* mice and stimulated with anti-CD3 and anti-CD28 for 2–3 days. The percent of nonviable CD4^+^ or CD8^+^ T cells is shown. Cells were obtained from one mouse per group and tested in quadruplicate. Data are representative of four separate experiments. **P < 0.*01*, ***P < 0.*001* (Mann-Whitney *U*-test).

We previously showed that *Drak2* ^*-/-*^ T cells exhibit enhanced susceptibility to death *in vivo*, which promotes resistance to type 1 diabetes and multiple sclerosis [2]. In addition, we found that following *in vitro* stimulation with anti-CD3 and anti-CD28, a greater proportion of *Drak2* ^*-/-*^ T cells were apoptotic compared to wildtype T cells (Fig 6a and 6b, left portion of graph). Although we did not observe differences in TGF-β signaling in the absence of *Drak2*, there may be alternative TGF-β-mediated effects on T cell survival. Thus, we sought to determine if the survival defect in *Drak2* ^*-/-*^ T cells compared to wildtype T cells was due to enhanced TGF-β signaling. To test this, we compared T cell survival between wildtype and *Drak2* ^*-/-*^ T cells that exhibit impaired TGF-β signaling due to expression of a dominant-negative TGF-β receptor II (*DNRII*) transgene. The *DNRII* transgene is a kinase-dead mutant that blocks signaling through the endogenous TGF-β receptor by competing for TGF-β binding [17]. Naïve CD4^+^ and CD8^+^ T cells were sorted from wildtype, *Drak2* ^*-/-*^, *DNRII*, and *DNRII*.*Drak2* ^*-/-*^ mice. The purified T cells were stimulated *in vitro* with anti-CD3 and anti-CD28. We found that even with the severe reduction in TGF-β signaling, there was an increase in the proportion of nonviable *DNRII*.*Drak2* ^*-/-*^ CD4^+^ (Fig 6a, right portion of graph) and CD8^+^ (Fig 6b, right portion of graph) T cells compared to *DNRII* CD4^+^ and CD8^+^ T cells. These data show that the enhanced death in the *Drak2* ^*-/-*^ T cells following *in vitro* stimulation is not due to increased TGF-β signaling, and suggest that alternative signaling pathways play a role.

## Discussion

Drak2 contributes to organ-specific autoimmune disease and is an ideal protein to target to treat these diseases without causing generalized immune suppression. Therefore, it is pertinent to understand the molecular and cellular mechanisms by which Drak2 functions in order to further comprehend the etiology of autoimmune disease. In addition, insight into the function of Drak2 is critical to validate it as a therapeutic target.

TGF-β is a multifunctional cytokine that controls many aspects of T cell behavior and elicits protective effects in several autoimmune diseases [13]. It has been suggested that Drak2 functions as a negative regulator of TGF-β signaling [12]. As TGF-β can inhibit proliferation, survival, and differentiation of T cells, enhanced TGF-β signaling in *Drak2* ^*-/-*^ T cells could contribute to the resistance to autoimmune disease in the *Drak2* ^*-/-*^ mice via one or more of these mechanisms. However, our data suggest that in primary T cells stimulated *in vitro*, Drak2 does not function as a negative regulator of this pathway. Smad2/3 signaling after TGF-β stimulation was not enhanced in *Drak2* ^*-/-*^ T cells compared to wildtype T cells. Importantly, the impact of TGF-β on T cell behavior was not enhanced in the absence of *Drak2* as evidenced by equal inhibition of naïve T cell proliferation, comparable effects on activated CD8^+^ T cell accumulation and survival, and similar induction of regulatory T cells in wildtype and *Drak2* ^*-/-*^ T cells.

The previous studies that suggested Drak2 negatively regulates TGF-β signaling were performed in tumor cell lines [12]. It is possible that Drak2 inhibits TGF-β in certain tumor cells, but not in primary T cells. Mutations that lead to tumorigenesis could facilitate a role for Drak2 regulation in TGF-β signaling. Thus, as reported in certain tumors, Drak2 may function to negatively regulate TGF-β signaling and promote tumorigenesis [12]. However, this is contrary to other reports that describe Drak2 as a tumor suppressor [18–20]. Therefore, the role of Drak2 in different types of tumors is also controversial and needs to be studied further. Interestingly, we have shown that *Drak2* ^*-/-*^ mice respond similarly to wildtype mice in various *in vivo* tumor models, again suggesting that the role of Drak2 in cell lines may not mimic its role under physiological conditions [7].

Furthermore, it is important to investigate the molecular mechanisms of Drak2 in primary T cells, as these are the cells relevant to the induction or resistance to autoimmunity. The importance of Drak2 specifically in T cells during autoimmunity was highlighted in our previous studies, which demonstrated that the resistance to disease in the mouse model of multiple sclerosis was due to *Drak2-*deficiency in T cells [2]. In addition, we found that the resistance to type 1 diabetes was also due to the absence of *Drak2* in T cells (TLH and MAM manuscript submitted).

Another possible explanation for the discrepancy between our results in primary T cells and the previous data in tumor cell lines is that during development, the *Drak2* ^*-/-*^ T cells may have compensated for the loss of Drak2 through modifications of alternate pathways involved in TGF-β regulation. For example, increased levels of Smad7, a negative regulator of TGF-β signaling [21], could mask alterations in TGF-β signaling in the absence of *Drak2*. Therefore, *Drak2* ^*-/-*^ T cells may exhibit altered signaling pathways that function differently compared to physiological conditions in wildtype T cells, which warrants further investigation.

Nevertheless, our data presented here indicate that TGF-β signaling is not enhanced in *Drak2* ^*-/-*^ T cells following *in vitro* stimulation. Consequently, Drak2 may not function as a negative regulator of TGF-β signaling in T cells, which are critical for the induction of autoimmunity. Therefore, further investigation of the potential molecular mechanisms by which Drak2 functions during autoimmune disease is required to gain insight into the etiology of these diseases.

## References

[1] McGargill MA, Wen BG, Walsh CM, Hedrick SM. A deficiency in Drak2 results in a T cell hypersensitivity and an unexpected resistance to autoimmunity. Immunity. 2004;21: 781–791. 10.1016/j.immuni.2004.10.008 15589167 PMC2792702

[2] McGargill MA, Choy C, Wen BG, Hedrick SM. Drak2 regulates the survival of activated T cells and is required for organ-specific autoimmune disease. J Immunol Baltim Md 1950. 2008;181: 7593–7605. 19017948 10.4049/jimmunol.181.11.7593 PMC2792703

[3] Ramos SJ, Hernandez JB, Gatzka M, Walsh CM. Enhanced T cell apoptosis within Drak2-deficient mice promotes resistance to autoimmunity. J Immunol Baltim Md 1950. 2008;181: 7606–7616. 19017949 10.4049/jimmunol.181.11.7606 PMC2709975

[4] Wang S, Welte T, McGargill M, Town T, Thompson J, Anderson JF, et al Drak2 contributes to West Nile virus entry into the brain and lethal encephalitis. J Immunol Baltim Md 1950. 2008;181: 2084–2091. 18641347 10.4049/jimmunol.181.3.2084 PMC2494872

[5] Ramos SJ, Hardison JL, Stiles LN, Lane TE, Walsh CM. Anti-viral effector T cell responses and trafficking are not dependent upon DRAK2 signaling following viral infection of the central nervous system. Autoimmunity. 2007;40: 54–65. 10.1080/08916930600996700 17364498

[6] Schaumburg CS, Gatzka M, Walsh CM, Lane TE. DRAK2 regulates memory T cell responses following murine coronavirus infection. Autoimmunity. 2007;40: 483–488. 10.1080/08916930701651139 17966037

[7] Edwards BA, Harris TL, Floersh H, Lukens JR, Zaki MH, Vogel P, et al Drak2 is not required for tumor surveillance and suppression. Int Immunol. 2015; 10.1093/intimm/dxu146 PMC4817074 25568303

[8] Sanjo H, Kawai T, Akira S. DRAKs, novel serine/threonine kinases related to death-associated protein kinase that trigger apoptosis. J Biol Chem. 1998;273: 29066–29071. 9786912 10.1074/jbc.273.44.29066

[9] Matsumoto M, Miyake Y, Nagita M, Inoue H, Shitakubo D, Takemoto K, et al A serine/threonine kinase which causes apoptosis-like cell death interacts with a calcineurin B-like protein capable of binding Na(+)/H(+) exchanger. J Biochem (Tokyo). 2001;130: 217–225. 11481038 10.1093/oxfordjournals.jbchem.a002975

[10] Newton RH, Leverrier S, Srikanth S, Gwack Y, Cahalan MD, Walsh CM. Protein kinase D orchestrates the activation of DRAK2 in response to TCR-induced Ca2+ influx and mitochondrial reactive oxygen generation. J Immunol Baltim Md 1950. 2011;186: 940–950. 10.4049/jimmunol.1000942 PMC3133617 21148796

[11] Mao J, Luo H, Han B, Bertrand R, Wu J. Drak2 is upstream of p70S6 kinase: its implication in cytokine-induced islet apoptosis, diabetes, and islet transplantation. J Immunol Baltim Md 1950. 2009;182: 4762–4770. 10.4049/jimmunol.0802255 19342653

[12] Yang K-M, Kim W, Bae E, Gim J, Weist BM, Jung Y, et al DRAK2 participates in a negative feedback loop to control TGF-β/Smads signaling by binding to type I TGF-βreceptor. Cell Rep. 2012;2: 1286–1299. 10.1016/j.celrep.2012.09.028 23122956

[13] Rubtsov YP, Rudensky AY. TGFbeta signalling in control of T-cell-mediated self-reactivity. Nat Rev Immunol. 2007;7: 443–453. 10.1038/nri2095 17525753

[14] McKarns SC, Schwartz RH. Distinct effects of TGF-beta 1 on CD4+ and CD8+ T cell survival, division, and IL-2 production: a role for T cell intrinsic Smad3. J Immunol Baltim Md 1950. 2005;174: 2071–2083. 15699137 10.4049/jimmunol.174.4.2071

[15] Sanjabi S, Mosaheb MM, Flavell RA. Opposing effects of TGF-beta and IL-15 cytokines control the number of short-lived effector CD8+ T cells. Immunity. 2009;31: 131–144. 10.1016/j.immuni.2009.04.020 19604492 PMC2765785

[16] Oh SA, Li MO. TGF-β: guardian of T cell function. J Immunol Baltim Md 1950. 2013;191: 3973–3979. 10.4049/jimmunol.1301843 24098055 PMC3856438

[17] Gorelik L, Flavell RA. Abrogation of TGFbeta signaling in T cells leads to spontaneous T cell differentiation and autoimmune disease. Immunity. 2000;12: 171–181. 10714683 10.1016/s1074-7613(00)80170-3

[18] Doherty GA, Byrne SM, Austin SC, Scully GM, Sadlier DM, Neilan TG, et al Regulation of the apoptosis-inducing kinase DRAK2 by cyclooxygenase-2 in colorectal cancer. Br J Cancer. 2009;101: 483–491. 10.1038/sj.bjc.6605144 19638987 PMC2720240

[19] Ye P, Zhao L, Gonda TJ. The MYB oncogene can suppress apoptosis in acute myeloid leukemia cells by transcriptional repression of DRAK2 expression. Leuk Res. 2013;37: 595–601. 10.1016/j.leukres.2013.01.012 23398943

[20] Kuwahara H, Nakamura N, Kanazawa H. Nuclear localization of the serine/threonine kinase DRAK2 is involved in UV-induced apoptosis. Biol Pharm Bull. 2006;29: 225–233. 16462023 10.1248/bpb.29.225

[21] Itoh S, ten Dijke P. Negative regulation of TGF-βreceptor/Smad signal transduction. Curr Opin Cell Biol. 2007;19: 176–184. 10.1016/j.ceb.2007.02.015 17317136

